# Evaluating the duration of antimicrobial therapy for the treatment of orthopedic hardware infections

**DOI:** 10.1128/spectrum.01269-24

**Published:** 2024-09-30

**Authors:** Rija Asim, Christian J. Fuchs, Nathan A. Summers

**Affiliations:** 1 Department of Medicine, University of Tennessee Health Science Center College of Medicine, Memphis, Tennessee, USA; 2 Department of Medicine, Division of Infectious Diseases, University of Tennessee Health Science Center, Memphis, Tennessee, USA; University of California San Diego, La Jolla, California, USA

**Keywords:** osteomyelitis, hardware infections, therapeutics

## Abstract

The optimal duration of antimicrobial therapy for orthopedic hardware infections is unclear. We identified 216 patients with orthopedic hardware infections, of whom 42 (19%) later had relapsed infection. Chronic suppressive antimicrobial therapy beyond 12 weeks was not significantly associated with lower odds of relapse.

**IMPORTANCE:**

There is debate about how long to continue antibiotics after initial treatment of bone and joint infections when hardware remains in place. This study found no benefit from continuing antibiotics longer than 12 weeks when trying to prevent recurrent infection.

## INTRODUCTION

The optimal duration of antibiotics for orthopedic hardware infections typically ranges from 6 to 8 weeks for most osteomyelitis (1, 2) and 12 weeks for prosthetic joint infections (PJIs) (3). Chronic suppressive antimicrobial therapy (CSAT), continuing antibiotics beyond the initial treatment course, has unclear benefits. Some suggest indefinite suppression for infections involving hardware (1, 4), but CSAT for more than 3 months may be unnecessary (5). This uncertainty regarding the need for and duration of CSAT is highlighted in the most recent guidelines (6, 7) calling for additional research.

In this retrospective study, we reviewed data from Regional One Health (ROH) in Memphis, TN, including all patients with osteomyelitis with retained hardware from traumatic fractures from 1 May 2019 to 1 May 2022. We performed manual chart reviews in the electronic health record (EHR) using a standardized case report form (CRF) to describe the management of orthopedic hardware infections.

## MATERIALS AND METHODS

All patients with osteomyelitis who attended a clinic visit at least once at the Infectious Diseases clinic at ROH from 1 May 2019 to 1 May 2022 were included for possible inclusion. Cases were identified from the EHR using The International Classification of Diseases, Ninth Revision, and The International Classification of Diseases, Tenth Revision, codes for osteomyelitis before manual review determined the presence of retained orthopedic hardware. Only those with confirmed retained hardware were included.

The primary outcome was whether the infection had relapsed at 1 year, defined as documented clinical or microbiologically confirmed infection at the same anatomical site as the initial infection. Clinical, laboratory, and outcome data were abstracted from the EHR in a standardized fashion using a CRF.

All statistical analyses were performed using SAS software, version 9.4 (SAS Institute, Cary, NC). A descriptive analysis was performed for all patients. A multivariable logistic regression, including predetermined patient and treatment factors, was performed. These factors were selected based on prior works suggesting potential risk factors for relapsed infection (5, 8–11). The use of rifampin was planned to be included in the analysis but had to be dropped from the final model due to low frequency of use and model stability. Other factors, such as hardware site and specific culture results, had too small numbers in certain categories, leading to unstable model results.

## RESULTS

The demographics of the patients and the treatments are described in Table 1. Of the 216 patients included, 42 (19%) had relapse. The average age was 45 years, 138 (64%) were male, and 117 (55%) identified as Black. The most common pathogen identified was methicillin-resistant *Staphylococcus aureus* (MRSA), median initial treatment duration was 6 weeks, 63% of patients had oral treatment, and rifampin was used for 24% of patients. Also, 42% of patients were given CSAT with an average duration of 52 weeks.

**TABLE 1:** Patient characteristics, *N* = 216

Characteristic	Overall (*N* = 216)	No relapse (*N* = 174)	Relapse (*N* = 42)
Age, years, mean (SD)	45 (16.67)	44 (16.45)	46 (17.73)
Race, *N* (%)			
Black	117 (54.93)	92 (53.80)	25 (59.52)
White	87 (40.85)	70 (40.94)	17 (40.48)
Other	9 (4.23)	9 (5.26)	0
Hispanic	14 (6.48)	14 (8.05)	0
Gender			
Male	138 (63.89)	106 (60.92)	32 (76.19)
Female	78 (36.11)	68 (39.08)	10 (23.81)
Diabetes mellitus, *N* (%)	25 (11.57)	17 (9.77)	8 (19.05)
Charlson Comorbidity Index ≥ 2, *N* (%)	89 (41.20)	70 (40.23)	19 (45.24)
Prior infection at the same site, *N* (%)	38 (17.59)	33 (18.97)	5 (11.90)
Hardware age <6 months, *N* (%)	121 (56.02)	95 (54.60)	26 (61.90)
Hardware site, *N* (%)			
Leg	97 (45.12)	72 (41.62)	25 (59.52)
Arm	21 (9.77)	20 (11.56)	1 (2.38)
Hip	4 (1.86)	4 (2.31)	0
Other	93 (43.26)	77 (44.51)	16 (38.10)
C-reactive peptide, median (IQR)			
First	3.4 (1.1, 11.4)	3.1 (1.1, 10.85)	4.7 (1.2, 11.9)
Last	0.8 (0.5, 2.0)	0.8 (0.5, 2.4)	0.6 (0.5, 1.0)
White blood cell count ≥12,000 cells/µL, *N* (%)	135 (63.08)	105 (61.05)	30 (71.43)
Culture results, *N* (%)			
Methicillin-sensitive *Staphylococcus aureus*	38 (17.59)	45 (28.30)	7 (17.07)
Methicillin-resistant *Staphylococcus aureus*	57 (26.39)	45 (28.30)	12 (29.27)
Coagulase-negative *Staphylococcus*	46 (21.29)	35 (22.01)	11 (26.83)
*Streptococcus* spp.	18 (8.33)	13 (8.18)	5 (12.20)
*Enterococcus*	12 (5.56)	7 (4.40)	5 (12.20)
Gram-negative	40 (18.52)	32 (20.13)	8 (19.51)
Other	64 (29.63)	46 (28.93)	18 (43.90)
Polymicrobial	57 (26.39)	39 (22.41)	18 (42.86)
Negative	16 (7.41)	15 (8.62)	1 (2.38)
Baseline resistance, *N* (%)			
Extended-spectrum beta-lactamase	5 (2.31)	4 (2.30)	1 (2.38)
Vancomycin-resistant *Enterococcus*	1 (0.46)	1 (0.57)	0
Initial treatment duration, weeks, median (IQR)	6 (6, 8)	6 (6, 8)	6 (6, 6)
Route of treatment, *N* (%)			
Oral	136 (63.26)	112 (64.74)	24 (57.14)
Intravenous	79 (36.74)	61 (35.26)	18 (42.86)
Use of rifampin, *N* (%)	52 (24.30)	45 (26.16)	7 (16.67)
Combined with a fluoroquinolone, *N* (%)	21 (9.81)	19 (11.05)	2 (4.76)
Suppressive therapy given, *N* (%)	91 (42.13)	72 (41.38)	19 (45.24)
Suppressive therapy duration, weeks, median (IQR)	52 (12, 52)	52 (16, 52)	24 (12, 52)
>12 weeks, *N* (%)	46 (21.30)	36 (20.69)	0 (23.81)
Indefinite duration, *N* (%)	17 (7.87)	12 (6.90)	5 (11.90)
Lost to follow-up, *N* (%)	64 (29.77)	57 (32.95)	7 (16.67)
Development of resistance, *N* (%)	2 (0.93)	2 (1.15)	0
Side effects from treatment, *N* (%)			
None	192 (88.89)	154 (88.51)	38 (90.48)
Gastrointestinal	17 (7.87)	13 (7.47)	4 (9.52)
*Clostridioides difficile* infection	3 (1.39)	3 (1.72)	0
Other	4 (1.85)	4 (2.30)	0

Table 2 provides information on characteristics affecting relapse. High white blood cell (WBC) and polymicrobial infection were significantly associated with relapse. The use of CSAT beyond 12 weeks did not affect relapse.

**TABLE 2:** Factors affecting relapsed infection at 1 year from completing initial antimicrobial treatment

Characteristic	No relapse	Relapse	Odds ratio (95% CI)	*P* value^*a*^
Age, mean (SD)	44 (16.45)	46 (17.73)	0.99 (0.94, 1.03)	0.50
Female, *N* (%)	68 (39.08)	10 (23.81)	0.79 (0.15, 4.22)	0.79
Hardware age < 6 months, *N* (%)	72 (41.62)	26 (61.90)	5.50 (0.57, 52.93)	0.14
Diabetes mellitus, *N* (%)	17 (9.77)	8 (19.05)	4.18 (0.30, 58.79)	0.29
WBC count ≥12,000 cells/µL, *N* (%)	105 (61.05)	30 (71.43)	15.64 (1.20, 204.57)	0.04
Polymicrobial infection, *N* (%)	39 (22.41)	18 (42.86)	8.57 (1.52, 48.49)	0.02
Suppressive antibiotics > 12 weeks, *N* (%)	36 (81.82)	10 (83.33)	1.63 (0.21, 12.40)	0.64

^*a*^ Calculated by multivariable logistic regression controlling for other variables listed in the table.

## DISCUSSION

In summary, this observational study found that of the 216 patients treated for osteomyelitis with retained hardware, 42 (19%) had relapse. In multivariable analysis, an elevated WBC count and the presence of a polymicrobial infection were independently associated with increased odds of relapse, while CSAT beyond 12 weeks was not.

While 20% of patients developing a relapsed infection was higher than the typical rate of 1–2% for most fracture repairs (12), it is closer to rates with open fractures, where infection rates historically range from 10% to 50% (8, 10, 13, 14). As a level 1 trauma center, many of the fractures were open fractures, which naturally carry a higher risk of developing infection. Unfortunately, the proportion of open fractures in our dataset was not available due to inconsistent documentation, leading to incomplete data.

Our primary aim was to assess the benefit of prolonged CSAT for the treatment of orthopedic hardware infections from traumatic fractures. Guidelines for MRSA infections suggest considering indefinite suppression when hardware remains (1), but a single study found no benefit in extending suppression beyond 3 months (5). We found that the odds of relapse were not significantly different when CSAT was continued beyond 12 weeks, consistent with their findings.

The only two factors in multivariable analysis that were significantly associated with increased odds of relapsed infection were a WBC count ≥12,000 cells/µL and the presence of a polymicrobial infection. Previous works evaluating inflammatory markers have found mixed results, with some finding an association with relapsed infection (14) and others not (8, 11). Polymicrobial infections have similarly been debated as a risk factor for relapse, with some studies finding higher rates of relapse (9) and others not (8). If a polymicrobial infection is a surrogate for the degree of contamination in open fractures, then our findings are consistent with other works finding greater contamination to be associated with relapse (10).

We unfortunately had low rates of relapse in the rifampin arm, limiting its inclusion in multivariable analysis. Guidelines for PJIs recommend the use of rifampin in combination therapy (4), but this is based on a single trial (15). A meta-analysis found that the greatest benefit from rifampin was when it was combined with fluoroquinolone, and that combination with other antibiotics had a minimal effect (16). While relapses were less common among those who were given adjunctive rifampin in our work, the overall use of rifampin was low, making statistical comparisons limited.

Our work has several limitations. There were more patients lost to follow-up before the planned treatment end date among those who did not have a relapse (33%) compared with those who had a relapse (17%). While we would expect patients who develop relapsed infections or complications related to treatment to return to the clinic, this disparity could potentially skew our findings if the majority of those lost to follow-up ended up having a relapse but were seen at a different facility. Also, because of our overall sample size, some patient factors had very small numbers, making statistical analysis limited. A larger, prospective trial could plan accordingly and increase recruitment numbers to ensure the evaluation of additional patient factors not able to be evaluated in our smaller study. Additionally, as a retrospective observational study, it is impossible to control for unmeasured confounders or determine causality. Therefore, our results should be viewed as practice-informing and hypothesis-generating rather than definitive.

A key question facing clinical providers managing orthopedic hardware infections is when it is safe to stop antimicrobials. While our work does not definitively answer this question, it adds to the growing body of literature that prolonged treatment courses for a variety of infections are not always better (17). Relapse rates for orthopedic hardware infections are high, and identifying who needs to be treated for how long is critical to improve patient outcomes while avoiding unnecessary antibiotics.
